# A nomogram with the peripheral blood neutrophil-to-lymphocyte ratio before treatment predicts the survival of patients with nasopharyngeal carcinoma

**DOI:** 10.3389/fonc.2025.1469191

**Published:** 2025-05-08

**Authors:** Huai-wen Zhang, Liu Feng, Hao-wen Pang, Teng-hua Yu, Tian-zhu Lu, Jin-gao Li, Bin Xu, Chun-ling Jiang

**Affiliations:** ^1^ Department of Radiation Oncology, Jiangxi Cancer Hospital & Institute, Jiangxi Clinical Research Center for Cancer, The Second Affiliated Hospital of Nanchang Medical College, Nanchang, China; ^2^ Department of Oncology, Huanggang Central Hospital, Huanggang, China; ^3^ Department of Oncology, The Affiliated Hospital of Southwest Medical University, Sichuan, China; ^4^ Key Laboratory of Personalized Diagnosis and Treatment of Nasopharyngeal Carcinoma, Medical College of Nanchang University, Nanchang, China

**Keywords:** nasopharyngeal carcinoma, neutrophil-to-lymphocyte ratio, overall survival, prognostic factors, nomograms

## Abstract

**Background:**

This study was performed to investigate the relationship of the pretreatment neutrophil count and neutrophil-to-lymphocyte ratio (NLR) with the prognosis of nasopharyngeal carcinoma (NPC), as well as to establish an NLR-related nomogram to predict survival in patients with NPC.

**Methods:**

In total, 747 patients with NPC were enrolled between January 2005 and January 2015 at our hospital. Kaplan–Meier survival analysis was used to evaluate overall survival (OS), progression-free survival (PFS), and distant metastasis-free survival (DMFS), with comparisons made using the log-rank test. Univariate and multivariate Cox regression analyses were conducted to identify independent risk factors for OS, PFS, and DMFS. The optimal NLR cut-off value was determined using receiver operating characteristic curve analysis. A nomogram model was then constructed and validated using R software (Version 3.6.0).

**Results:**

Among the 747 patients, N stage (P = 0.01, 0.042, 0.017) and NLR (P = 0.037) were identified as independent predictors of DMFS. Independent predictors of OS were sex (P = 0.024), age (P = 0.019), N stage (P = 0.006, 0.031, 0.002), American Joint Committee on Cancer (AJCC) stage (P = 0.003), adjuvant chemotherapy (P = 0.016), and NLR (P = 0.036). N stage (P = 0.001, 0.0221, 0.003), AJCC stage (P = 0.001), and NLR (P = 0.035) were also associated with PFS. The prognostic model showed good agreement with actual outcomes. Compared with the TNM staging system, the nomogram demonstrated superior accuracy and stability.

**Conclusions:**

In patients with NPC, an elevated pretreatment NLR was associated with poorer OS, PFS, and DMFS. The NLR-based nomogram provided more accurate survival prediction than clinical staging and may serve as a valuable tool in guiding prognosis and treatment planning.

## Highlights

- This study establish an NLR-related nomogram to predict the survival of patients with NPC.
- The nomogram with the NLR was better than TNM at predicting the OS of patients with NPC.

## 1 Introduction

Nasopharyngeal carcinoma (NPC) is a head and neck tumor with an uneven distribution, showing high incidence rates in southern China and Southeast Asia (1–5). Most cases of NPC are squamous cell carcinoma, characterized by low differentiation, high malignancy, and rapid growth and invasiveness because of its unique anatomical location. Early diagnosis of NPC is challenging, and approximately 80% of patients present with advanced complications at the time of diagnosis (6, 7). Because of its location, NPC is not easily treated with surgery. Currently, radiotherapy with or without chemotherapy is the first-line treatment for NPC (8–10). With advancements in imaging technology and improvements in chemotherapy equipment and regimens, the local control rate of NPC has improved significantly. Initially, 4%–10% of NPC cases are diagnosed with distant metastasis (7, 11); following treatment, the rate of distant metastasis rises to 15%–30% (12). This suggests that distant metastasis is the primary cause of treatment failure in NPC (13, 14). Clinical data have confirmed that lymph node involvement, metastasis, and TNM stage are among the most valuable prognostic factors for NPC (15). However, the prognosis is influenced by multiple variables, and the complexity of these factors means that the TNM staging system alone cannot comprehensively or accurately predict clinical outcomes. Patients with the same TNM stage often show marked differences in treatment efficacy (16). Therefore, identifying economical, convenient, and objective factors to supplement TNM staging is essential for predicting the prognosis of patients with NPC.

The clinical prognosis of patients with cancers and their response to therapy are directly related to immune cells. A growing number of studies have shown that inflammatory cells promote tumor growth and metastasis by altering the biological characteristics of tumor cells and activating stromal cells in the tumor microenvironment, including vascular endothelial cells, tumor-associated macrophages, and fibroblasts. Neutrophils can coordinate the immune response, activate inflammation, and secrete cytokines and inflammatory factors. These actions play an important role in the initiation and progression of tumors. Zhu (17) demonstrated that neutrophil progenitor cells in humans and mice promote tumor growth. Moreover, these cells are more abundant in the blood of patients with melanoma than in healthy individuals, suggesting that detection of neutrophil progenitor cells may serve as an early warning signal for tumors. Murakami (18) found that patients with gastric cancer who had higher levels of peripheral blood neutrophils responded poorly to chemotherapy and had a reduction in overall survival (OS) of 8 months on average relative to those with lower neutrophil levels.

The neutrophil-to-lymphocyte ratio (NLR) is defined as the ratio of neutrophils to lymphocytes in peripheral blood and serves as a composite index reflecting systemic immune status. In a study of pancreatic cancer, 206 patients were retrospectively analyzed (19). For patients with an NLR of ≥5, the median survival time (4 months) was significantly shorter than for those with an NLR of <5 (12 months), and an elevated NLR was associated with a poor prognosis (19). In another study assessing the prognostic value of the NLR in metastatic colorectal cancer, the NLR of 413 patients was retrospectively analyzed (20). Both OS and progression-free survival (PFS) were significantly shorter in patients with an NLR of ≥3 than in those with an NLR of <3. These findings highlight the prognostic value of NLR in malignant tumors.

In this study, we investigated the relationship between neutrophils, the NLR, and survival in patients with NPC. We evaluated the prognostic value of the NLR in this patient population, aiming not only to identify new prognostic indicators but also to provide a simple, practical tool for everyday clinical use to support clinicians.

## 2 Materials and methods

### 2.1 Study design and participants

From January 2005 to December 2015, a total of 747 patients with newly diagnosed NPC were enrolled. For cases diagnosed after 2008, clinical tumor staging was determined according to the American Joint Committee on Cancer (AJCC) 7th edition criteria; cases diagnosed before 2008 were re-staged according to the same criteria. The inclusion criteria were a complete medical history, physical examination, hematology and blood biochemistry, magnetic resonance imaging of the nasopharynx and neck, chest computed tomography, and abdominal ultrasound. All clinical data were randomly divided into a training set and a validation set in a 7:3 ratio.

### 2.2 Clinical data and follow-up

The following clinical data were collected: sex, age, T stage, N stage, clinical stage, induction chemotherapy, number of induction chemotherapy cycles, concurrent chemotherapy, number of concurrent chemotherapy cycles, adjuvant chemotherapy, number of adjuvant chemotherapy cycles, and pretreatment values of white blood cells, neutrophils, lymphocytes, platelets, and hemoglobin. Hematological tests were performed prior to admission.

The area under the receiver operating characteristic (ROC) curve was 0.56 (95% confidence interval [CI]: 0.50–0.62), indicating that the model had some discriminatory ability. The optimal cut-off value could be determined through ROC curve analysis to balance the model’s sensitivity and specificity. In this study, guided by clinical practice, high sensitivity (≥80%) was set as the optimization goal. The NLR cut-off value was 1.886, and the median neutrophil count was 3.71 × 10^9^/L. At this threshold, the sensitivity was 82.76%, the specificity was 27.58%, and the overall accuracy (coincidence rate) was 34.00%. The T stage was categorized into two groups: T1+T2 and T3+T4. The N stage was divided into three groups: N0, N1+N2, and N3a+N3b. The clinical stage was grouped into stage I+II and stage III+IV.

The last follow-up was conducted on 1 March 2020, with a median follow-up duration of 47 months (until death or last follow-up). Patients were followed every 3 months during the first 3 years and every 6 months during years 4 and 5. The follow-up period ranged from 3 to 141 months. OS and distant metastasis-free survival (DMFS) were the primary endpoints, and PFS was a secondary endpoint. OS was calculated from the time of initial diagnosis to either the last follow-up or death of any cause. DMFS was measured from diagnosis to the last follow-up in patients who developed metastasis at distant sites. PFS was measured from diagnosis to the last follow-up in patients with metastasis at any new site or recurrence of lesions.

### 2.3 Statistical analysis

All data were analyzed using SPSS Version 23.0 (SPSS Inc., Armonk, NY, USA) and R software Version 3.6.1 (R Foundation for Statistical Computing, Vienna, Austria). Two-thirds of the patients were randomly assigned to the training group and one-third to the validation group. The chi-square test or independent-samples t-test was used to describe and compare the characteristics of the two groups. Kaplan–Meier analysis was used to calculate survival probabilities, and survival differences between groups were assessed using the log-rank test. Univariate and multivariate Cox regression analyses were conducted to identify independent risk factors for OS, PFS, and DMFS. ROC analysis was used to assess model sensitivity and specificity.

The discriminative ability of the nomogram and its predictive performance compared with the TNM stage were assessed using the concordance index (C-index). A value of 0.5 indicates random predictability, while a value of 1.0 indicates perfect predictability. The bootstrap self-sampling technique was used for internal and external validation of the nomogram. The nomogram model was divided into three groups based on the total score. Kaplan–Meier survival curves were generated, and log-rank tests were used to evaluate the model’s risk stratification performance. A two-tailed P value of <0.05 was considered statistically significant.

## 3 Results

### 3.1 Patient baseline characteristics

The characteristics of the training and validation groups are shown in **Table 1**. Among the 747 patients, 117 (15.8%) were in the early stage (I+II) and 630 (84.3%) were in the advanced stage (III+IV) according to the AJCC criteria. A total of 126 (16.8%) patients received only radiotherapy, while 622 (83.2%) received chemotherapy. Specifically, 12 (1.9%) patients received neoadjuvant chemotherapy, concurrent chemotherapy, and adjuvant chemotherapy; 282 (45.4%) received neoadjuvant chemotherapy plus concurrent chemoradiotherapy; 183 (29.4%) received neoadjuvant chemotherapy plus adjuvant chemotherapy; and 144 (23.3%) received concurrent chemoradiotherapy plus adjuvant chemotherapy. The chemotherapy regimen consisted of three cycles of cisplatin and 5-fluorouracil as induction chemotherapy every 3 weeks, two cycles of cisplatin for concurrent chemoradiotherapy every 3 weeks, and three cycles of paclitaxel as adjuvant chemotherapy every 3 weeks. A total of 119 (15.9%) patients developed distant metastasis. There were 114 (15.3%) deaths, with 87 (76.3%) patients dying of recurrence or metastasis of NPC and 27 (23.7%) dying of other related diseases. No patients died during treatment.

**Table 1:** Baseline characteristics.

Variable	Patients data (N=747)
Age, (n%)	
≤60	632 (84.61)
>60	115 (15.39)
Gender, n (%)	
male	517 (69.21)
female	230 (30.79)
T-stage, n (%)	
T1-T2	197 (26.37)
T3-T4	550 (73.63)
N-stage, n (%)	
N0	70 (9.37)
N1-N2	550 (73.63)
N3a-N3b	127 (17.00)
AJCC-stage, n (%)	
I -II	117 (15.66)
III-IV	630 (84.34)
Chemotherapy, n (%)	
Yes	622 (83.27)
No	125 (16.73)
Neoadjuvant chemotherapy, n (%)	
Yes	486 (65.06)
No	261(34.94)
Concurrent chemotherapy, n (%)	
Yes	409 (54.75)
No	338 (45.25)
Adjuvant chemotherapy, n (%)	
Yes	231 (30.92)
No	516 (69.08)
Neoadjuvant chemotherapy cycle, n (%)	
<2	249 (51.23)
≥2	237 (48.77)
Concurrent chemotherapy cycle, n (%)	
<2	224 (54. 77)
≥2	185 (45.23)
Adjuvant chemotherapy cycle, n (%)	
<2	92 (39.82)
≥2	139 (60.18)
Leukocyte, n(%)	
normal	643 (86.08)
abnormal	104 (13.92)
Monocytes, n (%)	
normal	706 (94.51)
abnormal	41 (5.49)
Platelets, n (%)	
normal	655 (87.68)
abnormal	92 (12.32)
Hemoglobin, n (%)	
normal	681 (91.16)
abnormal	66 (8.84)
NLR, n(%)	
High (≥1.886)	391 (52.34)
Low (<1.886)	356 (47.66)
Neutrophils, n (%)	
High (>3.71)	371 (49.66)
Low (≤3.17)	376 (50.34)
Metastasis	
Yes	119 (15.93)
No	628 (84.07)
Dead	
Yes	114 (15.26)
No	633 (84.73)

### 3.2 Kaplan–Meier survival analysis of neutrophil count, NLR, and N stage

**Figure 1:**
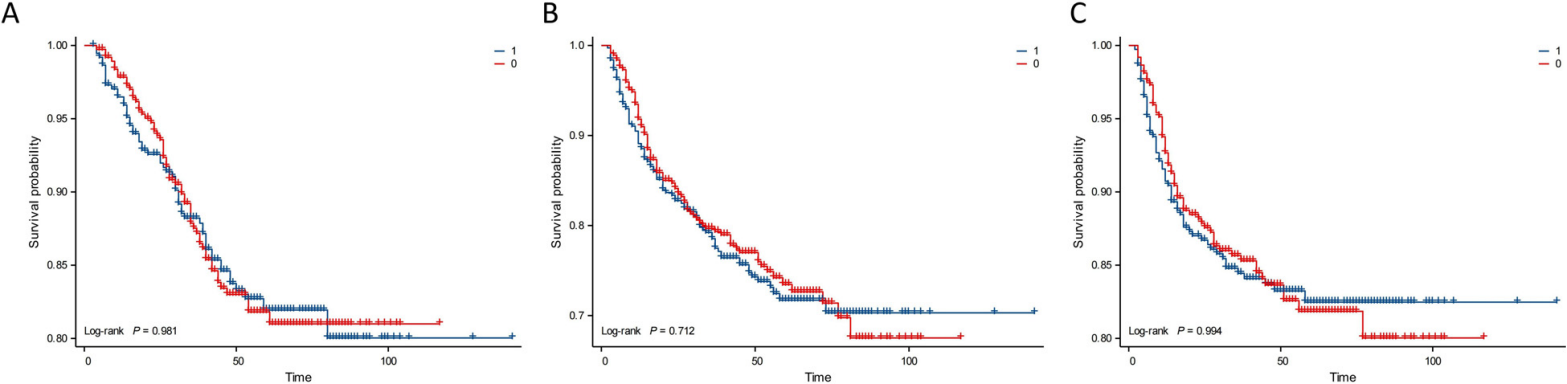
Kaplan–Meier analysis based on neutrophil subgroups. Group 1: neutrophils ≥ 3.71 × 10^9^/L; Group 0: neutrophils < 3.71 × 10^9^/L. **(A)** OS. **(B)** PFS. **(C)** DMFS.

**Figure 2:**
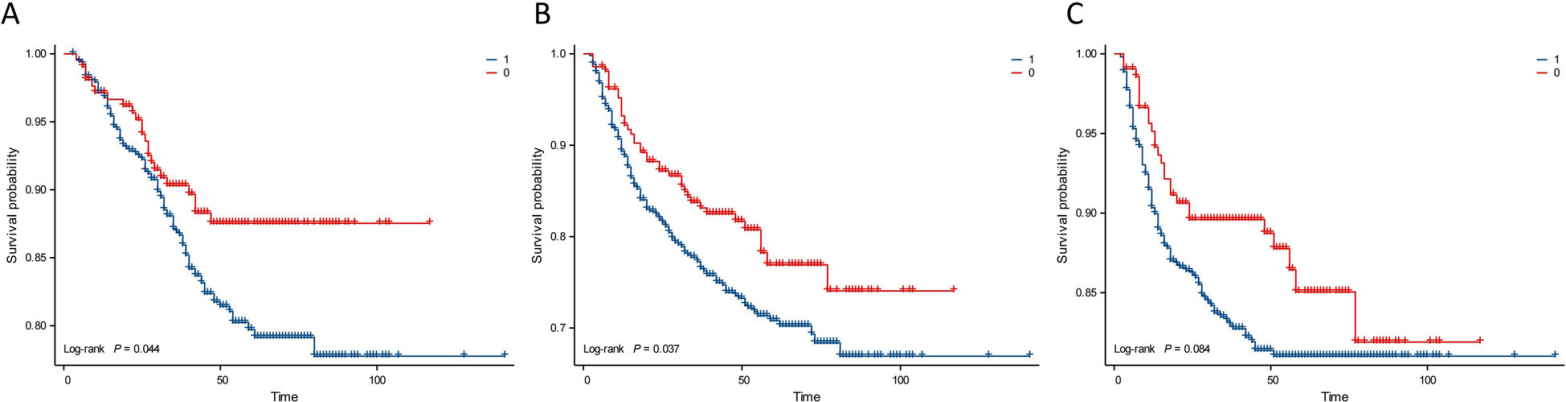
Kaplan–Meier analysis based on NLR. Group 1: NLR ≥ 1.886; Group 0: NLR < 1.886. **(A)** OS. **(B)** PFS. **(C)** DMFS.

**Figure 3:**
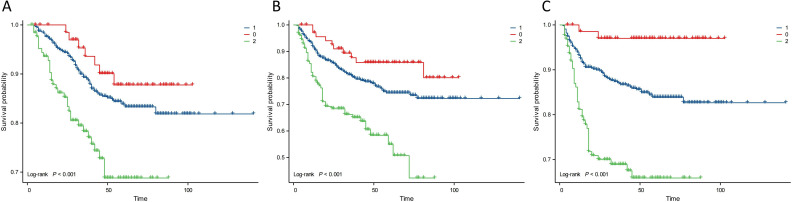
Kaplan–Meier analysis based on N stage. Group 0: N0; Group 1: N1+N2; Group 2: N3a+N3b. **(A)** OS. **(B)** PFS. **(C)** DMFS.

Kaplan–Meier analysis showed that the neutrophil count had no effect on OS, PFS, or DMFS (**Figures 1A–C**). The 3- and 5-year OS and PFS differed significantly between the low and high NLR groups (P < 0.05), although there were no significant differences in DMFS (P > 0.05). Patients with a lower NLR had significantly higher 3- and 5-year OS and PFS (**Figures 2A–C**). The N stage was significantly associated with OS, PFS, and DMFS (**Figures 3A–C**).

### 3.3 Univariate analysis

In the univariate analysis, the NLR (P = 0.026), N stage (P < 0.001), sex (P = 0.038), age (P = 0.009), T stage (P = 0.021), AJCC stage (P = 0.002), and neoadjuvant chemotherapy (P = 0.010) were significantly associated with OS. The NLR (P = 0.015), N stage (P < 0.001), sex (P = 0.036), age (P = 0.021), and AJCC stage (P < 0.001) were significantly correlated with PFS. The NLR (P = 0.020) and N stage (P < 0.001) were associated with DMFS (**Table 2**).

**Table 2:** Univariate analysis results of factors influencing OS, PFS, and DMFS in patients with NPC.

Project indicators	Variable	Regression coefficient	Standard error	P	HR (95%CI)
OS	gender			0.038	1.731 (1.030, 2.848)
	Age	-0.814	0.531	0.009	0.921 (0.604, 2.154)
	T-stage	-0.426	0.198	0.021	0.817 (0.369, 0.894)
	N-stage	-0.348	0.215	<0.001	2.624 (0.422, 3.921)
	AJCC-stage	-1.374	0.468	0.002	1.368 (0.142, 2.879)
	NLR	-0.934	0.116	0.026	1.859 (1.065, 3.245)
	Neutrophil	-0.108	0.224	0.172	1.074 (0.705, 1.635)
	adjuvant chemotherapy	1.021	0.964	0.545	1.151 (0.730,1.813)
	neoadjuvant hemotherapy	-0.356	0.21	0.01	0.653 (0.387,0.795)
PFS	gender			0.036	1.623 (1.210, 1.945)
	Age	-0.744	0.342	0.021	0.637 (0.352, 0.759)
	N-stage	-0.371	0.224	<0.001	1.402 (0.777, 1.397)
	AJCC-stage	-0.321	0.687	<0.001	0.960 (0.716, 1.287)
	NLR	-0.826	0.198	0.015	1.562 (1.088, 2.243)
	Neutrophil	-1.215	1.368	0.173	1.042 (1.795, 5.810)
DMFS	N-stage	-0.442	0.637	<0.001	3.229 (1.795,5.810)
	NLR	-0.623	0.869	0.02	1.316 (0.861,2.010)
	Neutrophil	-0.981	2.012	0.171	0.976 (1.399,2.099)

### 3.4 Multivariate analysis

**Table 3** shows that sex (P = 0.024), age (P = 0.019), N stage (P = 0.006, 0.031, 0.002), AJCC stage (P = 0.003), adjuvant chemotherapy (P = 0.016), and NLR (P = 0.036) were significant predictors of OS. N stage (P = 0.01, 0.042, 0.017) and NLR (P = 0.037) were prognostic factors for DMFS. N stage (P = 0.001, 0.0221, 0.003), AJCC stage (P = 0.001), and NLR (P = 0.035) were significantly associated with PFS. These results suggest that a high NLR is associated with poorer OS, PFS, and DMFS in patients with NPC. In other words, a high NLR predicts a worse prognosis.

**Table 3:** Multivariate Cox regression analysis results of factors influencing OS, DMFS, and PFS in patients with NPC.

Project indicators	Variable	Regression coefficient	Standard error	P	HR (95%CI)
**OS**	gender	-0.541	0.239	0.024	0.582(0.364, 0.931)
	Age	-0.544	0.231	0.019	0.581(0.369, 0.914)
	N-stage (N0)			0.006	
	N-stage(N1-N2)	-0.924	0.429	0.031	0.397(0.171, 0.919)
	N-stage(N3a-N3b)	-0.691	0.218	0.002	0.501(0.327, 0.768
	AJCC-stage	-1.374	0.468	0.003	0.253(0.101, 0.633)
	neoadjuvant hemotherapy	0.326	1.231	0.193	0.757(0.498, 1.151)
	adjuvant chemotherapy	0.524	0.216	0.016	1.688(1.105, 2.580)
	NLR	-0.398	0.190	0.036	0.671(0.463, 0.975)
	NLR	0.378	0.179	0.035	1.46 (1.03, 2.08)
**PFS**	N-stage (N0)			0.001	
	N-stage (N1-N2)	0.416	0.333	0.021	1.52 (0.79, 2.92)
	N-stage (3a-N3b)	1.040	0.356	0.003	2.83 (1.41, 5.69)
	AJCC-stage	0.994	0.307	0.001	2.70 (1.48, 4.94)
	N-stage (N0)			0.010	
**DMFS**	N-stage(N1-N2)	-0.924	0.429	0.042	0.431(0.201, 0.971)
	N-stage(N3a-N3b)	-0.691	0.218	0.017	0.512(0.358, 0.868)
	NLR	-0.398	0.190	0.037	0.643(0.441, 0.865)

### 3.5 Construction of nomogram survival prediction model for NPC

**Figure 4:**
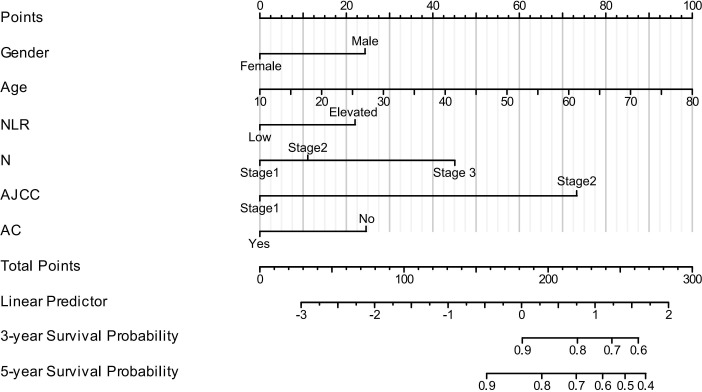
Nomogram model for predicting 3-year and 5-year OS in patients with NPC. (In N stage, Stage 1 = N0, Stage 2 = N1–N2, and Stage 3 = N3a–N3b. In AJCC stage, Stage 1 = Stage I–II and Stage 2 = Stage III–IV. AC, adjuvant chemotherapy. For NLR, low = <1.886, elevated = ≥1.886).

The nomogram was created using R software. It was developed based on the significant prognostic factors identified in the multivariate analysis, with the goal of predicting 3- and 5-year OS. The nomogram incorporated key prognostic indicators, including the NLR, sex, age, N stage, clinical stage, and adjuvant chemotherapy. Among these, clinical stage contributed most significantly to survival prediction, followed by N stage, sex, age, NLR, and adjuvant chemotherapy. The model was constructed using the training group by integrating all factors affecting OS as determined by Cox multivariate regression analysis, as shown in **Figure 4**. The nomogram indicated that AJCC stage had the greatest impact on survival in patients with NPC, followed by N stage, sex, age, NLR, and adjuvant chemotherapy.

### 3.6 Nomogram development and validation

**Figure 5:**
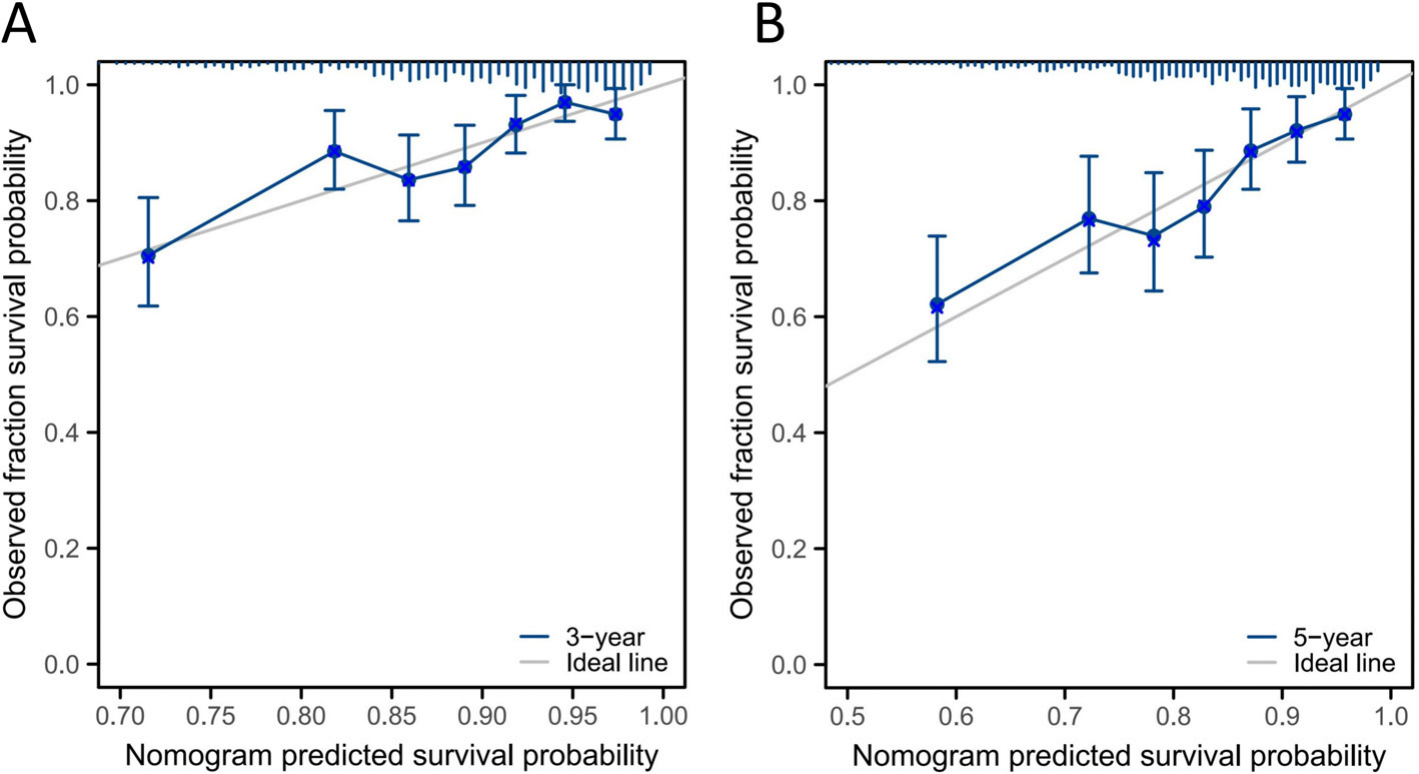
Calibration curves of the nomogram. **(A)** 3-year OS. **(B)** 5-year OS.

**Figure 5** shows the calibration curves for the 3- and 5-year OS probabilities of patients with NPC in the validation group. The curves demonstrate acceptable consistency between the predicted and actual survival outcomes at both time points.

The C-index of the nomogram model was compared with that of the TNM staging system. In the training group, the C-index was 0.700 (95% CI: 0.676–0.724), which was significantly higher than that of the TNM staging system (C-index = 0.627, 95% CI: 0.581–0.673; P < 0.001). In the validation group, the C-index was 0.670 (95% CI: 0.594–0.730), also significantly higher than that of the TNM staging system (C-index = 0.619, 95% CI: 0.561–0.633), as shown in **Table 4**. These results indicate that the model outperformed the TNM staging system in predicting the prognosis of patients.

**Table 4:** Comparison of nomogram model and TNM staging system in predicting 3-year OS probability.

Model	TNM Staging System	Nomogram	P
C-index (Training Dataset)	0.627	0.700	<0.001
C-index (Validation Dataset)	0.619	0.670	<0.001

**Figure 6:**
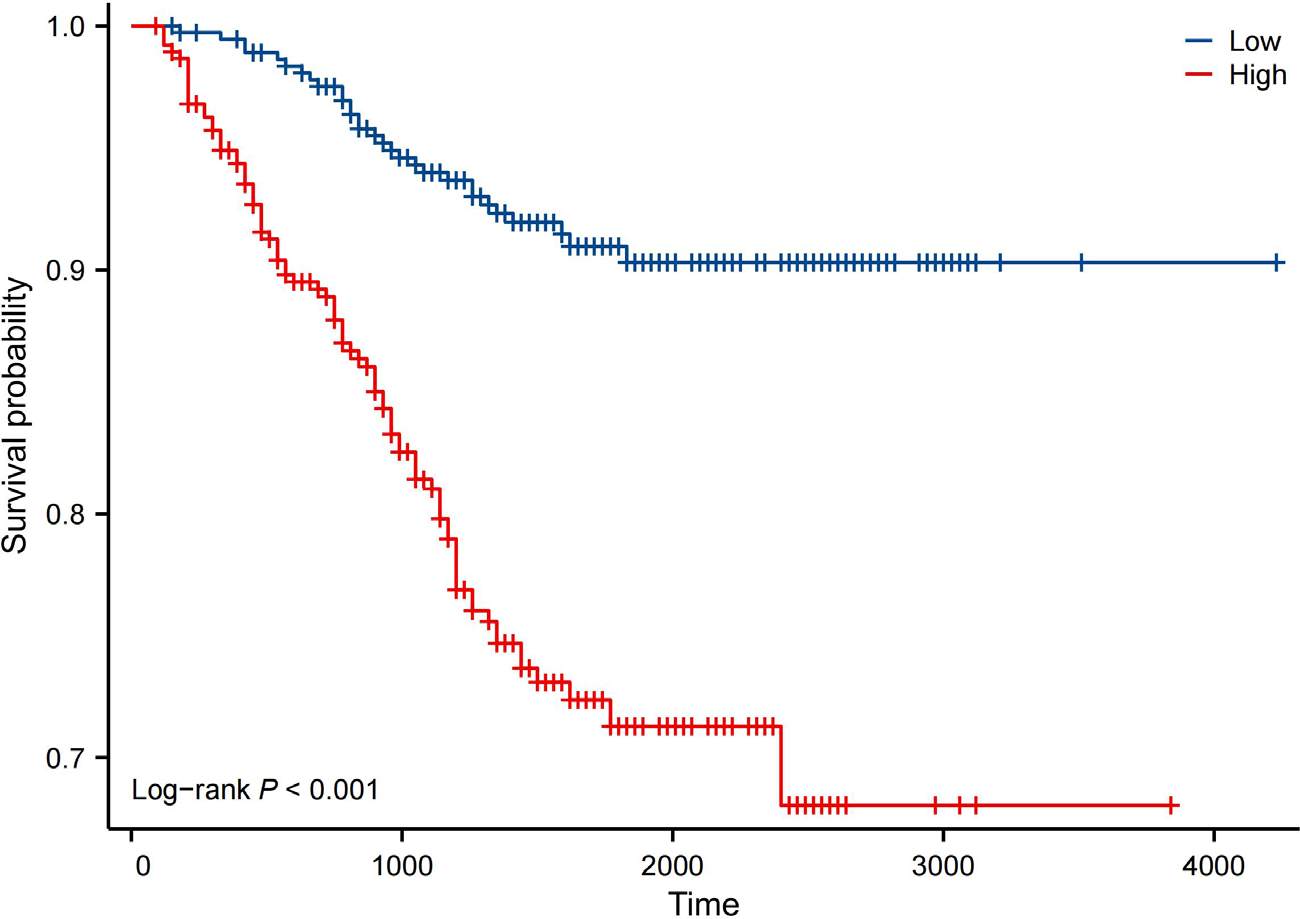
OS in high- and low-risk groups based on nomogram prediction results (days).

To evaluate the ability of the nomogram to stratify patients by risk, patients in the training group were divided into high- and low-risk groups based on the nomogram scores. The log-rank test indicated significant differences in survival time between the subgroups (P < 0.05). The Kaplan–Meier survival curve is shown in **Figure 6**.

## 4 Discussion

This study investigated the effects of sex, age, N stage, clinical stage, adjuvant chemotherapy, and NLR on OS and DMFS in patients with NPC. By combining the NLR with N staging, a nomogram prediction model was constructed, offering a new perspective on the prognostic value and clinical significance of NLR in NPC. The nomogram, based on these independent prognostic factors, demonstrated superior predictive ability, accuracy, and stability compared with the traditional TNM staging system. Among the factors included, the nomogram showed that the AJCC stage had the greatest impact on NPC survival, followed by N stage, sex, age, NLR, and adjuvant chemotherapy—findings consistent with the results reported by Tang (21).

Researchers have shown that the tumor microenvironment is a key factor influencing the genesis and progression of tumors, playing both immunosuppressive and immune escape roles that impact therapeutic efficacy (22, 23). Tumor-associated macrophages play a vital role in each step of tumor progression and metastasis (24, 25). The NLR in peripheral blood before treatment is a typical indicator of the systemic inflammatory response. Studies on pancreatic, colorectal, liver, and lung cancers have demonstrated that an elevated NLR is significantly correlated with a poor tumor prognosis (26–29). A meta-analysis by Zhao et al. (30), which included 14 studies and 6,693 patients, showed that an NLR above the threshold was significantly associated with OS and PFS (OS: hazard ratio [HR] 1.760, 95% CI 1.470–2.120; PFS: HR 1.850, 95% CI 1.430–2.390). Miao et al. (31) evaluated the prognostic value of the NLR in 406 patients with non-metastatic NPC and found that the NLR was an independent prognostic factor for PFS (HR 1.674, 95% CI 1.006–2.784, P = 0.047) and OS (HR 4.143, 95% CI 2.111–8.129, P = 0.000). A logistic regression analysis by Ye et al. (32) showed that the monocyte-to-eosinophil ratio before treatment and the NLR after treatment were independent predictors of OS in patients with advanced NPC; both the monocyte-to-eosinophil ratio and the NLR before and after treatment were independent prognostic factors for DMFS in this population. Li (33) retrospectively analyzed clinical data from 342 patients with NPC and found through Cox multivariate analysis that a high NLR was significantly correlated with OS and PFS. Lv et al. (34) significantly improved the accuracy of prognosis and survival prediction in NPC by constructing a comprehensive predictive model that included age, TNM stage, immunoinflammatory index, and NLR. These recent findings are generally consistent with our results. In our study, Kaplan–Meier analysis and univariate and multivariate Cox analyses showed that patients with a high NLR had worse OS, DMFS, and PFS than those with a low NLR. The NLR was an independent prognostic factor and demonstrated predictive value for survival assessment. Additionally, Setakornnukul et al. (35) and Song et al. (36) each confirmed the value of the NLR as an independent prognostic factor through retrospective cohort studies, identifying NLR cut-off values of 3 and 2.02, respectively (compared with our cut-off value of 1.889, likely due to the larger sample size in our study). This further supports the importance of the NLR in NPC prognosis evaluation.

Neutrophils can regulate the tumor microenvironment and promote the production of various pro-tumor growth factors and proteases. For example, neutrophils can release matrix metalloproteinase-9 and vascular endothelial growth factor, triggering tumor migration. Matrix metalloproteinase-9 can accelerate the release of vascular endothelial growth factor and promote tumor initiation and progression (37, 38). Sagiv (39) identified two types of neutrophils with different densities in peripheral blood—high-density neutrophils and low-density neutrophils. The latter possess immunosuppressive functions that enable rapid tumor cell growth. As the tumor progresses, the number of circulating low-density neutrophils sharply increases. By consuming arginine in the tumor microenvironment, these low-density neutrophils inhibit T-cell activation and promote tumor progression by impairing antigen recognition, which may be a key factor limiting the effectiveness of current immunotherapies. Lymphocytes play an essential role in the immune response and are a crucial component of anti-tumor immunity. A reduction in lymphocytes indicates immune dysfunction and weakened anti-tumor activity, creating favorable conditions for tumor growth, invasion, and metastasis (40). However, clinical studies examining the relationship between the number of peripheral blood neutrophils or lymphocytes and tumor prognosis remain limited. Chen (41) found that OS was worse in patients with high peripheral neutrophil counts (>4 × 10^9^/L) than in those with lower counts. In our study, we found no correlation between the peripheral neutrophil count and OS, DMFS, or PFS in patients with NPC. However, the NLR was significantly correlated with these survival measures. This finding suggests that the NLR may serve as a marker of the systemic inflammatory response and immune status. An elevated NLR indicates an imbalance between pro-tumor and anti-tumor inflammatory responses, enhancing tumor erosion and potentially leading to progression, metastasis, and ultimately a poor prognosis (42). The body’s immunosuppressive state affects the tumor microenvironment and immune response, influencing tumor development and prognosis in patients with NPC (43).

Although NPC has a 5-year survival rate of up to 80% with treatment of the primary tumor, metastasis remains a major challenge (44). The high metastatic rate of NPC increases the risk of death and severely impacts the prognosis, making it a persistent and difficult problem for the medical community to overcome (45). Therefore, treatment strategies should be adjusted accordingly for high-risk metastatic NPC, with systemic therapy as the main approach, supplemented by enhanced local treatment when necessary. In this study, we found that the pretreatment NLR was an independent prognostic factor for OS, PFS, and DMFS. As a hematological marker, the NLR offers several advantages over other tests—it is economical, convenient, safe, and non-invasive. These benefits suggest that the NLR may aid in understanding the invasive behavior of NPC and in predicting a high risk of metastasis. Clinically, for patients with a high NLR, and when the patient’s condition allows, more aggressive and comprehensive treatment strategies should be considered to prolong OS and improve the prognosis. This finding provides a new direction for clinical research into NPC treatment and supports the use of the NLR as a biomarker for guiding future clinical management of the disease.

Accurate survival prediction and the implementation of individualized treatment strategies for patients with NPC are pressing issues in clinical practice. Many researchers have developed nomogram models to predict NPC outcomes, and these have been shown to be more accurate than the TNM staging system for survival assessment (46). However, the NLR is rarely included in these models as an independent prognostic factor. Sun (47) included 353 patients and constructed a nomogram using age, N stage, and Epstein–Barr virus DNA levels to predict 3- and 5-year survival rates in NPC, but they did not incorporate the NLR or clinical stage. In our study, we included 747 patients to construct a nomogram prediction model based on 6 factors: sex, age, N stage, AJCC clinical stage, adjuvant chemotherapy, and NLR. The C-index values for the training and validation groups were 0.700 and 0.670, respectively, indicating good discrimination. According to the calibration curve, the nomogram also demonstrated good calibration. Patients were scored using the model and, based on the total score, the training group was divided into two risk groups. The Kaplan–Meier survival curve showed significant differences between these groups, demonstrating that the model had a strong risk stratification effect. Compared with the TNM staging system, the nomogram exhibited a higher C-index and showed good stability and accuracy in predicting OS probabilities. These results may offer useful insights for clinicians: patients with a higher pretreatment NLR might require more intensive treatment. Of course, this remains speculative, and the appropriate treatment model needs to be confirmed through prospective clinical trials.

This study had several limitations. First, it was a retrospective, single-center clinical study. The development and validation of our models were conducted internally, without external validation. Future large-scale, prospective, multi-center clinical trials—including in non-endemic populations—are needed to validate our findings. Second, this study did not collect all potentially relevant prognostic data for NPC, such as Epstein–Barr virus levels, lactate dehydrogenase levels, body mass index, high-sensitivity C-reactive protein levels, and tumor volume. Third, there is currently no standardized cut-off value for the NLR; in this study, the optimal threshold determined by ROC analysis was used as the cut-off.

## 5 Conclusions

In patients with NPC, a high NLR was associated with poorer OS, PFS, and DMFS. The NLR is an inexpensive and readily accessible biomarker that may help oncologists estimate the prognosis in patients with NPC.

## Data Availability

The original contributions presented in the study are included in the article/supplementary material. Further inquiries can be directed to the corresponding authors.
